# A Prognostic Model of Differentiated Thyroid Cancer Based on Up-Regulated Glycolysis-Related Genes

**DOI:** 10.3389/fendo.2022.775278

**Published:** 2022-04-22

**Authors:** Min Wu, Deng-jie Ou-yang, Bo Wei, Pei Chen, Qi-man Shi, Hai-long Tan, Bo-qiang Huang, Mian Liu, Zi-en Qin, Ning Li, Hui-yu Hu, Peng Huang, Shi Chang

**Affiliations:** ^1^ Department of General Surgery, Xiangya Hospital Central South University, Changsha, China; ^2^ National Clinical Research Center for Geriatric Disorders, Xiangya Hospital, Changsha, China; ^3^ Clinical Research Center for Thyroid Disease in Hunan Province, Xiangya Hospital, Changsha, China; ^4^ Hunan Provincial Engineering Research Center for Thyroid and Related Diseases Treatment Technology, Xiangya Hospital, Changsha, China

**Keywords:** thyroid cancer, prognosis, glycolysis-related genes, recurrence, decision tree.

## Abstract

**Objective:**

This study aims to identify reliable prognostic biomarkers for differentiated thyroid cancer (DTC) based on glycolysis-related genes (GRGs), and to construct a glycolysis-related gene model for predicting the prognosis of DTC patients.

**Methods:**

We retrospectively analyzed the transcriptomic profiles and clinical parameters of 838 thyroid cancer patients from 6 public datasets. Single factor Cox proportional risk regression analysis and Least Absolute Shrinkage and Selection Operator (LASSO) were applied to screen genes related to prognosis based on 2528 GRGs. Then, an optimal prognostic model was developed as well as evaluated by Kaplan-Meier and ROC curves. In addition, the underlying molecular mechanisms in different risk subgroups were also explored *via* The Cancer Genome Atlas (TCGA) Pan-Cancer study.

**Results:**

The glycolysis risk score (GRS) outperformed conventional clinicopathological features for recurrence-free survival prediction. The GRS model identified four candidate genes (ADM, MKI67, CD44 and TYMS), and an accurate predictive model of relapse in DTC patients was established that was highly correlated with prognosis (AUC of 0.767). *In vitro* assays revealed that high expression of those genes increased DTC cancer cell viability and invasion. Functional enrichment analysis indicated that these signature GRGs are involved in remodelling the tumour microenvironment, which has been demonstrated in pan-cancers. Finally, we generated an integrated decision tree and nomogram based on the GRS model and clinicopathological features to optimize risk stratification (AUC of the composite model was 0.815).

**Conclusions:**

The GRG signature-based predictive model may help clinicians provide a prognosis for DTC patients with a high risk of recurrence after surgery and provide further personalized treatment to decrease the chance of relapse.

## Introduction

Thyroid cancer is one of the most common endocrine malignancies, with an incidence that has substantially increased worldwide over the last several decades (1–3). Differentiated thyroid carcinoma (DTC) is the most common pathological subtype, including papillary thyroid carcinoma (PTC), follicular thyroid carcinoma (FTC), and Hürthle cell carcinoma (4). Generally, DTC has an excellent prognosis due to its indolent features and a better survival rate than other carcinomas; however, disease ultimately recurs in approximately 5–21% of DTC patients (5). In DTC patients who experience recurrent disease, surgical treatment is generally required, and reoperation poses additional medical costs and significant morbidity compared to the initial surgery. Therefore, preventing recurrence in DTC patients can reduce the deteriorations in quality of life related to reoperation (6–8).

According to previous reports, age, lymph node (LN) metastasis, tumor size, extrathyroidal extension (ETE), tumor multiplicity, and extranodal extension (ENE) are known risk factors for recurrence of DTC (9). Thus, the American Thyroid Association (ATA) management guidelines have proposed a clinicopathological risk stratification system based on those risk factors, which subdivides patients into high-risk and low-risk cohorts, providing prognostic and predictive information to facilitate clinical decisions. Unfortunately, since most prognostic signatures lack reproducibility due to individual heterogeneity, the current guidelines are unable to effectively predict recurrence in patients with DTC.

Our previous studies confirmed that metabolic reprogramming and aberrant expression of glycolysis-related genes have been characterized as hallmarks of DTC (10). A high rate of aerobic glycolysis, known as the “Warburg effect”, has been gradually confirmed to occur in various cancers, and treatment with novel therapeutic targets can be used as an effective anticancer strategy (11). Our previous studies also found that aerobic glycolysis is active in DTC, promoting thyroid tumorigenesis, proliferation and invasion (12). Additionally, mitochondrial glycerophosphate dehydrogenase (MGPDH), the key enzyme connecting oxidative phosphorylation (OXPHOS) and glycolysis, promotes Warburg metabolism in thyroid cancer and can be effectively targeted by metformin (13). Studies have shown that high lactate production due to abnormal glycolytic activity promotes tumor angiogenesis and invasion, which may play a role in suppressing immune cells against cancer and promoting tumor recurrence (14, 15). Therefore, targeting glycolysis is a promising strategy for cancer treatment. In addition, a glycolysis-related prognostic score for the prediction of prognosis and chemosensitivity of pancreatic ductal adenocarcinoma has shown better performance than conventional methods (16). However, limited studies have systematically investigated the metabolic status and its prognostic value in patients with DTC. The association between the genetic characteristics of glycolysis and the heterogeneity of DTC has never been reported.

In this study, a glycolysis-related prognostic signature was developed based on expression profiling data in patients with DTC from several datasets. Histopathology and cellular experiments were used to investigate the utility of a simplified protein signature. In addition, the underlying molecular mechanisms in different risk subgroups were also explored. This study aimed to clarify the relationship between glycolysis and DTC, as well as to determine whether this prognostic signature can be used to detect a group of patients with DTC who have a high risk of recurrence.

## Materials and Methods

### Patients and Datasets

To identify the expression levels of glycolysis-related genes (GRGs) in DTCs, we retrospectively analyzed the gene expression profiles and clinical parameters of DTCs patients from 6 public cohorts, including 5 datasets (GSE33630, GSE60542, GSE58545, GSE35570, and GSE27155) from GEO and 1 dataset (TCGA-THCA) from TCGA. We downloaded the RNA-seq data of 510 cancer samples and 58 normal samples from TCGA, only patients meet the following criteria were included: 1) R0 surgical margins, 2) intact recurrence status, and 3) recurrence time > 30 days. Patients who were treated with RAI were also included in the analysis. Overall, a total of 379 patients were included in our study (**Supplemental Table 1**). Patients from those 5 GEO datasets (n=400, **Supplemental Table 2**) were enrolled to form the validation cohort of differentially expressed GRGs, while, patients from TCGA-THCA were randomly divided into training set (n=191), validation set (n=188) and test set (n=379) for model validation.

### Candidate GRGs Selection and Signature Establishment

According to previous reports, 2528 protein-coding genes from GeneCards database were selected as GRGs (**Supplemental Table 3**) (17, 18). Then, we performed gene set variation analysis (GSVA) in upregulated GRGs (log2FC ≥ 1 and FDR < 0.05), and GSVA score for each sample was defined as the glycolytic carcinogenicity score (19). The candidate GRGs were screened from those upregulated GRGs that co-expressed in TCGA-THCA, 5 GEO datasets, and functioned as prognosis-related genes. Then, prognosis-related DEGs were imported into LASSO regression to construct the most stable glycolysis-related risk score (GRS) as follows:


$$GRS=\sum_{i}^{n}Coef\left(gene_{i}\right)*Expr\left(gene_{i}\right)$$

After the glycolysis-related gene signature (quantified by GRS) was established, univariate and multivariate Cox proportional hazards models were used to determine independent prognostic factors, thereafter, the independent prognostic factors, which were identified by multivariate Cox proportional hazards regression analysis, were used to create a composite risk score and displayed as a nomogram. Moreover, Schoenfeld residuals test were used to determine the proportional hazards assumption of the nomogram. Except for using ROC and calibration curves to measure the nomogram, we also performed decision curve analysis (DCA) to assess the clinical net benefit.

### Mechanism Exploration

Differentially expressed genes (*limma*, |logFC|≥1 and FDR<0.05) between the GRS-high and GRS-low groups were screened and analyzed for GO and KEGG enrichment. We further analyzed the infiltration extent of 22 immune cells in different risk populations using the *ESTIMATE* and *ssGSEA* algorithm, as well as the correlation between GRS genes and immune cell infiltration *via* TIMER tool (20). In particular, the ESTIMATE algorithm estimates stromal and immune scores to predict the level of infiltrating stromal and immune cells based on their specific gene expression profile (21). For validation of each possible mechanism, we performed a pan-cancer analysis *via* GSCALite portal. Overall, 10 cancers including bladder urothelial carcinoma (BLCA), breast invasive carcinoma (BRCA), esophageal carcinoma (ESCA), head and neck squamous cell carcinoma (HNSC), kidney renal clear cell carcinoma (KIRC), kidney renal papillary cell carcinoma (KIRP), liver hepatocellular carcinoma (LIHC), lung adenocarcinoma (LUAD), lung squamous cell carcinoma (LUSC), and stomach adenocarcinoma (STAD) were analyzed.

### Cell Culture and Plasmid Transfections

The DTC cancer cell lines TPC-1, BCPAP, and K1 and the normal thyroid Nthy-ori 3-1 cell line used in this study were provided by the Institute of Medical Sciences at Xiangya Hospital. All cell lines were cultured in a cell culture incubator at 37°C and 5% CO_2_ according to our previous study (10). Blank plasmids or siRNAs targeting the four GRGs were manufactured by Jikai Company (Shanghai, China). Cells were transiently transfected with blank plasmid or siRNA using Lipofectamine 2000 (Invitrogen) according to the manufacturer’s instructions.

### Western-Blot

Western blotting was performed as previously described (10) with Abs targeting ADM (ab190819, Abcam), CD44 (ab189524, Abcam), MKI67 (ab92742, Abcam) and TYMS (15047‐1‐AP, Proteintech).

### Cell Proliferation

For the cell proliferation assay, after transfection for 72 hours, the BrdU incorporation efficiency of cells was measured using the Cell Proliferation Assay kit (Roche, 11669915001) according to the manufacturer’s instructions.

### Statistical Analyses

Statistical analyses were performed using R software (version 3.6.1). The ESTIMATE package was used to calculate the immunoreactivity of the samples. The LASSO algorithm was performed *via* glmnet package. Survival analysis, and optimal cut-off values were performed and obtained through the survival and survminer packages. The rms, pROC and ggDCA package were used to plot calibration, ROC curves and DCA curves. The nomogram was plotted using the regplot package. The chi-square test was used to assess clinical correlation. All statistical tests with p-values less than 0.05 were considered significant if not otherwise stated.

## Results

### Schematic Diagram of the Study Design

**Figure 1:**
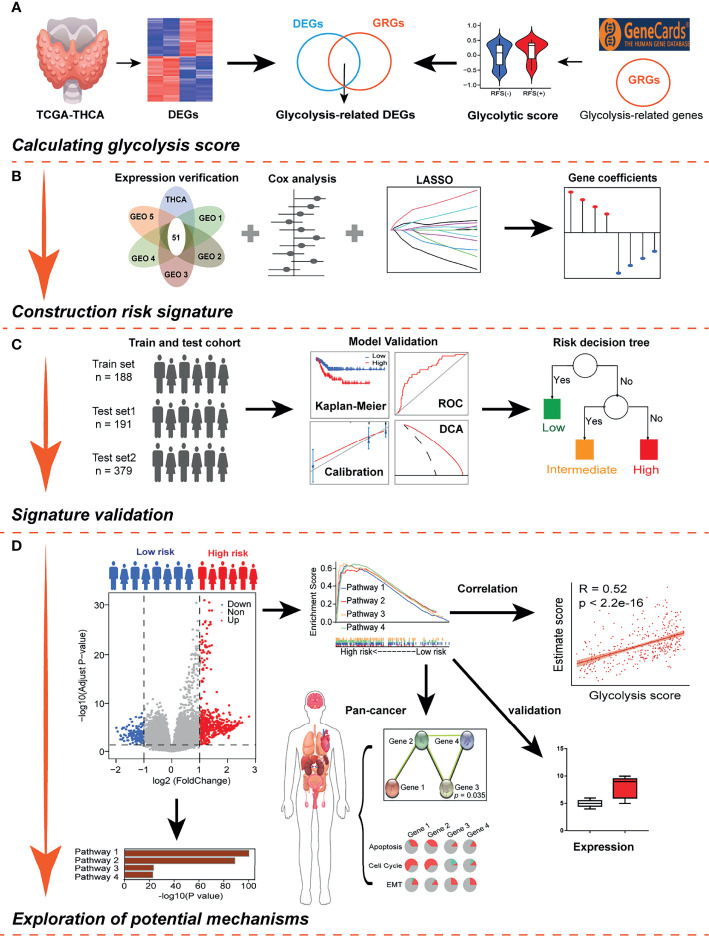
Schematic workflow of the study design. **(A)** Glycolysis was identified as an important risk factor for recurrence-free survival (RFS) in differentiated thyroid carcinoma (DTC). **(B)** Combined methods were used to establish a robust glycolysis-related gene signature for prognosis. **(C)** The prognostic value of the gene signature was validated in different cohorts, and a clinical decision tree was generated. **(D)** Exploring potential molecular mechanisms using pan-cancer analysis and cellular experiments.

First, glycolysis was identified as an important risk factor affecting relapse in DTC patients. Then, a combination of univariate Cox regression analysis and the LASSO algorithm was used to screen for robust glycolysis-related genes and create a glycolysis-related gene signature to predict recurrence and survival in DTC patients. Subsequently, the prognostic value of this gene signature was validated using the training and validation cohorts. In addition, model assessments were used to further evaluate its prognostic ability, and a decision tree was constructed to improve risk stratification for recurrence based on GRSs and other clinicopathological variables. Finally, histopathological examination and cellular experiments were used to explore potential molecular mechanisms in the high-risk group (**Figure 1**).

### Glycolysis Levels in Tumor Tissues Are Significantly Associated With Poor Prognosis in DTC Patients

**Figure 2:**
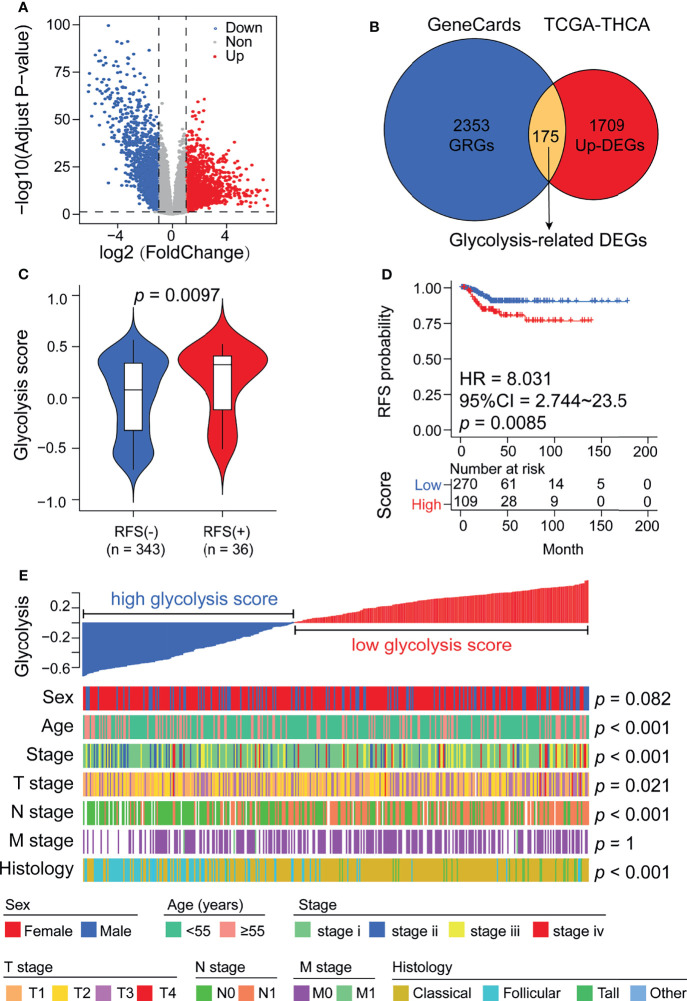
Glycolysis scores are associated with thyroid cancer recurrence and various clinicopathological features. **(A)** Volcano plot showing DEGs between normal thyroid and cancer samples in the TCGA cohort. **(B)** Venn diagram demonstrating the screening methods for 175 candidate glycolysis-related DEGs. **(C)** Glycolysis scores were significantly elevated in patients who relapsed after surgery. **(D)** Kaplan–Meier analysis showing that patients with higher glycolysis scores exhibited worse RFS. **(E)** A heatmap depicting the correlations between glycolysis scores and clinicopathological features.

To understand the correlation between aberrant expression of GRGs and the prognosis of DTC, we downloaded the RNA-seq data and matched clinical information of 379 DTC patients from TCGA. In our results, we identified 3258 differentially expressed genes (DEGs), including 1709 upregulated genes and 1549 downregulated genes (**Figure 2A**). Meanwhile, we screened 2353 glycolysis-related genes (GRGs) from the GeneCards website, 175 of which were selected as candidate genes by taking the intersection between DEGs and GRGs (**Figure 2B** and **Supplemental Figure 1**). Glycolysis scores based on 175 GRGs were used to assess the glycolysis level in tumor tissue. We found that the glycolysis scores in RFS (+) DTC were significantly higher than those in RFS (-) DTC (p < 0.05, **Figure 2C**). Moreover, using ROC curve analysis, we set the optimal cut-off value for glycolysis scores to 0.582 and found that DTC patients with high glycolysis scores exhibited worse prognosis (**Figure 2D**). Furthermore, glycolysis scores were found to be significantly associated with the clinicopathological features of DTC, including diagnosis age (p < 0.001), clinical stage (p < 0.001), T stage (p = 0.021), N stage (p < 0.001), and histology grading (p < 0.001) (**Figure 2E**). Besides, when we compared the levels of glycolysis scores between different clinical subgroups using ANOVA tests (**Supplemental Figure 2**). We found that overall glycolysis scores trended upwards with disease progression (AJCC stage). And patients with positive lymph node metastases had significantly higher glycolysis scores than those without lymph node metastases. Among the different histological subgroup types, the glycolysis score was significantly higher in the Tall-cell subgroup than in the other subgroups. However, the difference in glycolysis scores between the elderly and the young was not significant. This may be related to statistical methods.

### A GRS Model Based on Four Targeting Genes Has Potential for Predicting Recurrence in DTC Patients

**Figure 3:**
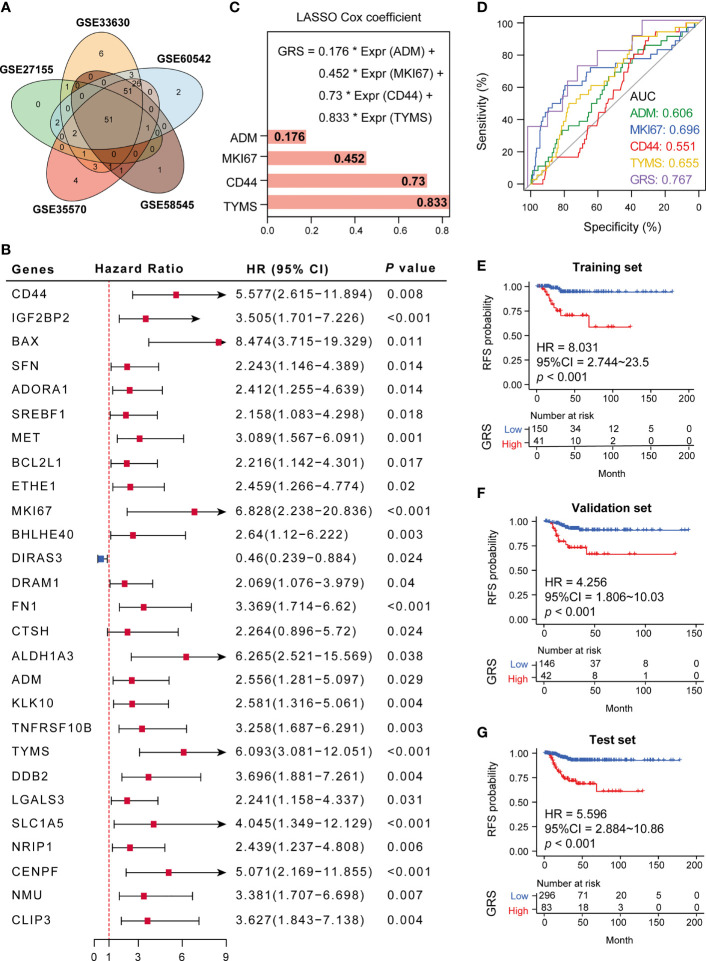
Establishment of a glycolysis-related gene signature. **(A)** Fifty-one GRGs were co-differentially expressed in 5 GEO cohorts (GSE33630, GSE60542, GSE58545, GSE35570 and GSE27155). **(B)** Twenty-seven differential glycolytic genes have prognostic value. **(C)** The Cox algorithm was used to construct a 4-gene glycolytic risk score (GRS). **(D)** ROC curve for DTC recurrence by glycolysis-related genes between patients with or without recurrence in the combined or respective GRG. **(E–G)** Kaplan–Meier curves of 4 glycolysis-related genes in the training, validation and test sets. *p < 0.05.

We next validated the expression of 175 GRGs in five GEO cohorts (GSE33630, GSE60542, GSE58545, GSE35570 and GSE27155), and 51 GRGs were confirmed to be upregulated in all cohorts (**Figure 3A**). However, only 27 GRGs were further confirmed to be significantly associated with RFS in DTC patients (**Figure 3B**). Next, the LASSO Cox algorithm was conducted to screen for robust glycolysis-related prognostic biomarkers. We finally constructed glycolysis risk score (GRS) predictive models based on four targeting genes (ADM, CD44, MKI67 and TYMS), which were highly upregulated in tumor samples, including papillary and follicular thyroid cancers (**Supplemental Figure 3**). The GRS was calculated *via* the combination GRG panel, GRS = (0.176×expression value of ADM) + (0.452×expression value of MKI67) + (0.73×expression value of CD44) + (0.833×expression value of TYMS) (**Figure 3C**). ROC curve analysis indicated that our GRS model has good predictive value for recurrence in DTC patients (AUC=0.739) (**Figure 3D**). Meanwhile, validation of the model showed that GRS was significantly elevated in RFS (+) patients compared to RFS (-) patients in the training, validation and test sets (**Figures 3E–G**).

### Increased Expression of the Four Targeting Genes Promotes Cell Proliferation and Is Significantly Related to Lymph Node Metastasis in DTC Patients

**Figure 4:**
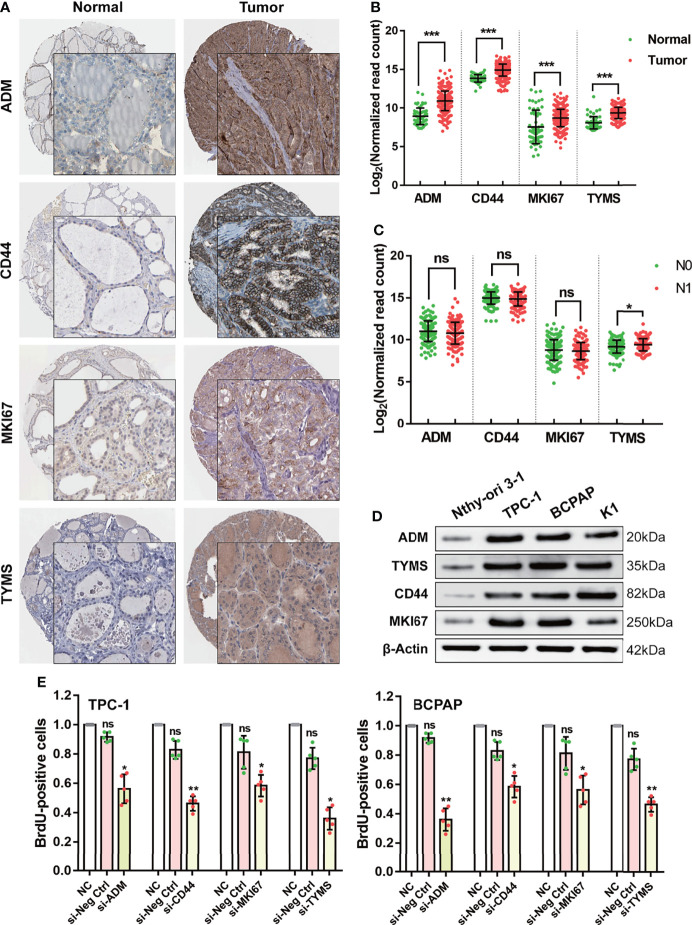
Validating the function of the four GRGs *in vivo* and *in vitro*. **(A)** Protein expression levels of the four GRGs in DTC and normal tissues in The Human Protein Atlas database. **(B)** Comparison of the expression of the four GRGs between DTC and normal tissues. **(C)** Comparison of the expression of the four GRGs in tumors with lymph node metastasis (N1) and without lymph node metastasis (N0). **(D)** Western blot detection of the four GRGs in normal thyroid cells (Nthy-ori 3-1) and DTC cell lines (TPC-1, BCPAP, K1). **(E)** DTC cells (TPC-1, BCPAP) were transfected with blank plasmid or siRNA targeting the four GRGs, and cell proliferation was assessed using a BrdU cell proliferation assay kit. Significance was tested by t-test, ***p<0.001, **p<0.01, *p<0.05, ns indicates no significance.

According to previous reports, the functions of CD44, ADM, TYMS, and MKI67 are primarily in promoting the Warburg effect and tumor growth in various cancers, while the function of those four genes in DTC has rarely been reported (**Supplemental Table 4**). Based on the available data from the HPA database (https://www.proteinatlas.org/), we observed high expression of CD44, ADM, TYMS, and MKI67 in tumor tissues compared to normal thyroid tissue (**Figure 4A**). The same results were confirmed using the data from TCGA (**Figure 4B**). Moreover, expression of TYMS in tumors with lymph node metastasis (N1) was higher than in tumors without (N0) (**Figure 4C**).

To elucidate the cellular function of the four targeting genes, we cultured four DTC cell lines and assessed changes in cell proliferation after alterations in their expression. We chose TPC-1 and BCPAP as cellular models since all four targeting genes are overexpressed in these lines (**Figure 4D**). Cell proliferation was determined by performing BrdU incorporation assays. In this study, silencing the four GRGs with siRNA resulted in a 40–70% decrease in cell proliferation in the two DTC cell lines (**Figure 4E**).

### The Four GRGs Involved in Remodeling of the Tumor Immune Microenvironment

**Figure 5:**
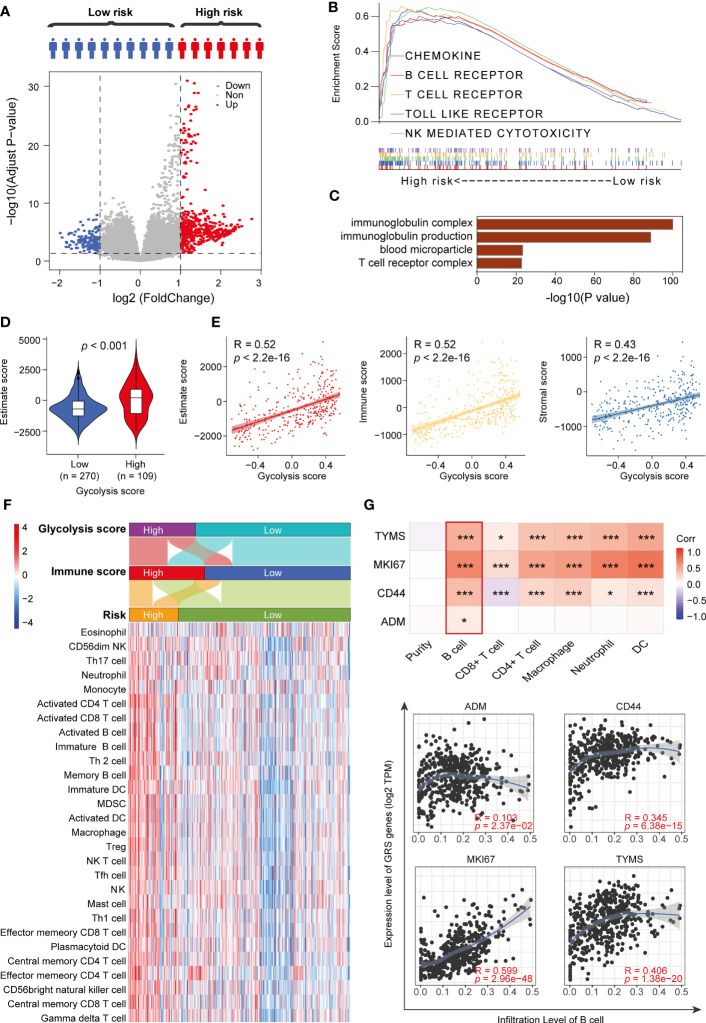
High-risk patients exhibit immune pathway activation. **(A)** Volcano plot showing DEGs between high-risk and low-risk patients. **(B)** GSEA results for the high-risk group. **(C)** GO terms for upregulated DEGs. **(D)** Immune status scores of different risk groups. **(E)** Correlation analysis of glycolysis scores and immune scores. **(F)** ssGSEA algorithm to assess the degree of immune cell infiltration in different risk groups. **(G)** TIMER algorithm to assess the correlation between GRS and the degree of immune cell infiltration. *p < 0.05 and ***p < 0.01.

To clearly clarify the molecular mechanisms of the four GRGs that may be involved, we divided the DTC patients into a low-GRS group and a high-GRS group. Using the limma algorithm, we identified 159 downregulated genes and 494 upregulated genes in the high-GRS group (**Figure 5A** and **Supplemental Table 5**), and those genes were primarily related to the activation of the immune signaling pathway (**Figures 5B, C** and **Supplemental Figure 4**). Next, we compared the distribution of immune scores in different GRS groups. The results revealed that the high-GRS group exhibited higher immunoreactivity and worse prognosis (**Figure 5D** and **Supplemental Figure 5**). In addition, we found that immunoreactivity scores were significantly correlated with glycolysis scores (**Figure 5E**). Next, we assessed the level of immune cell infiltration in different immune score groups, glycolysis score groups and risk groups using the *ssGSEA* algorithm. The results showed that the high-risk group population was also clustered into both the high glycolysis score group and the high immune score group, and the high-risk group displayed a higher level of immune cell infiltration (**Figure 5F**). Finally, we analyzed the correlation between the GRS genes and the degree of infiltration of the six immune cells using the TIMER algorithm. We found that CD44, MKI67 and TYMS were significantly associated with all six types of cells. In particular, the expression of GRS genes was positively correlated with the degree of B-cell infiltration in all of them. In addition, we explored the mutations of four GRS genes in thyroid cancer, their regulatory roles in ten classical tumor pathways, and their interactions (**Supplemental Figures 6**–**8**). We found that all GRS genes showed different degrees of mutation in thyroid cancer and were closely related to the cell proliferation cycle and epithelial-mesenchymal transition (EMT). Overall, our results suggest that the level of glycolysis may affect the patients’ immune status and thus the prognosis of thyroid cancer patients (**Figure 5G**).

### The Four GRGs Model Exhibited a Generalizability Signature in Pan-Cancer

**Figure 6:**
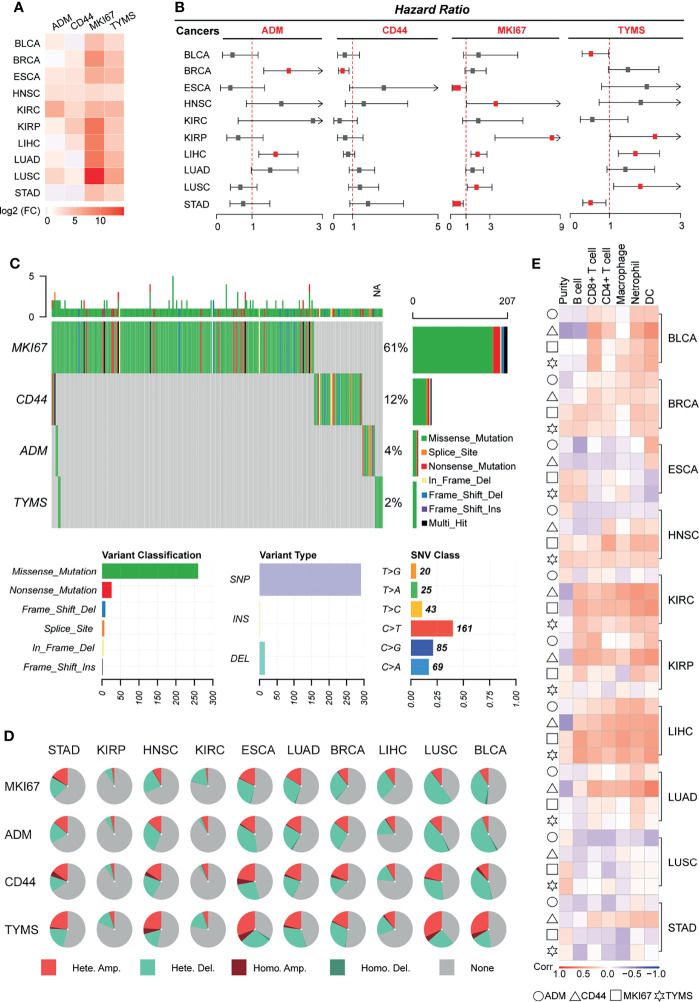
Validating the generalizability of the GRS predictive models across cancers. Ten types of tumors were used for pan-cancer analysis, and the pan-cancer RNA-Seq data were downloaded from TCGA. **(A)** Heatmap displaying the mRNA levels of GRS genes in different cancers. **(B)** Univariate Cox regression analysis of the association between the risk of recurrence and GRS gene mRNA levels. **(C)** Waterfall plot showing the SNV mutation signatures of four GRS genes. **(D)** Pie charts displaying the CNV mutations of four GRS genes. **(E)** Correlation of the expression of four GRS genes with immune cell infiltration.

In the pan-cancer analysis, we found that expression levels of the GRS model were significantly upregulated in all 10 cancer tissues (**Figure 6A** and **Supplemental Table 6**). Similarly, we observed that gene expression levels in the GRS model were strongly associated with recurrence-free survival for most tumors (**Figure 6B** and **Supplemental Table 7**).

Single nucleotide variant analysis revealed that MKI67 was the most frequently mutated gene (61%) among the four genes, missense mutation was the most common type of mutation, and C-base mutation to T-base was the most frequent base mutation (**Figure 6C**). We also explored the copy number variation of GRS genes across cancers. The results showed that GRS genes exhibited amplification and deletion in most cancers, with BLCA, ESCA and LUSC ranking highest in the frequency of GRS gene variants (**Figure 6D**). In the analysis of the degree of immune cell infiltration, we found that the mRNA expression level of the GRS genes was highly correlated with the degree of infiltration of six immune cells in BLCA, KIRC, and LIHC. In particular, LIHC was the tumor with the highest degree of correlation (**Figure 6E**). Besides, based on the genetic model, we calculated the GRS score for pan-cancer. The effect of GRS score on the prognosis and recurrence of pancytopenia was also examined using Kaplan-Meier analysis (**Supplemental Figure 9**). The results showed that GRS score for most tumors such as, GRS in BLCA, ESCA, KIRC, KIRP, LIHC and LUAD still showed as a risk factor for tumor outcome. High GRS scores were associated with poorer prognosis (*p*<0.05). Overall, our results suggest that GRS model may also be prognostic and therapeutic targets for other cancers.

### The Excellent Prognostic Predictive Efficacy of the GRS Model in DTC Patients Compared to Traditional Features

**Figure 7:**
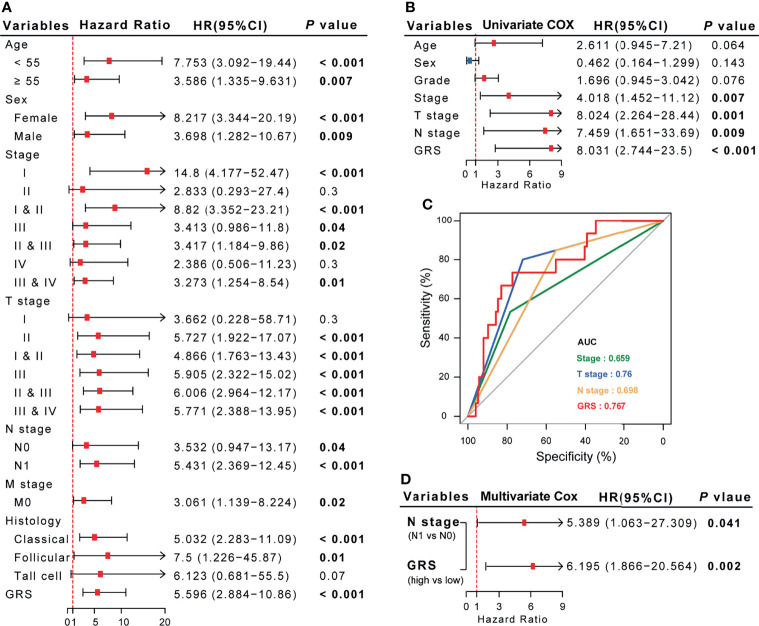
The comprehensive risk factor database for recurrence in DTC patients was established based on GRS and traditional features. **(A, B)** Univariate Cox regression analysis of the association between clinicopathological features, GRS and RFS. **(C)** Comparison of the diagnostic efficacy of GRS with traditional clinical indices *via* ROC curve analysis. **(D)** Multivariate Cox regression analysis demonstrated that N stage and GRS are independent risk factors for RFS in DTC patients.

According to previous reports, age, lymph node metastasis (LNM), tumor size, extrathyroidal extension (ETE), tumor multiplicity, and extranodal extension (ENE) are known risk factors for the recurrence of DTC (22). Consistent with the results of successive studies, we found that GRS also GRG also plays a role in most clinical subgroups. Besides, in this study (**Figure 7A**), stage, T stage, N stage and GRS were significantly correlated with the recurrence of DTC (**Figure 7B**). However, when comparing the predictive performance of GRS to traditional prognostic indicators, we observed better prognostic predictive efficacy of the GRS model for DTC patients than for traditional features. ROC curve analysis showed that the AUC of GRS was 0.739, while the AUC of sex, age, stage and grade was 0.431, 0.565, 0.515 and 0.634, respectively (**Figure 7C**). Furthermore, we compared the sensitivity and accuracy of the discriminatory efficacy for the GRS model in the first, third, and fifth years. As shown in **Supplemental Figure 10**, the AUCs of the GRS model in the first, third and fifth years were 0.774, 0.751 and 0.744, respectively.

### Integrated Model Constructed Using GRS and N-Stage Optimizes the Risk Stratification and RFS in DTC Patients

**Figure 8:**
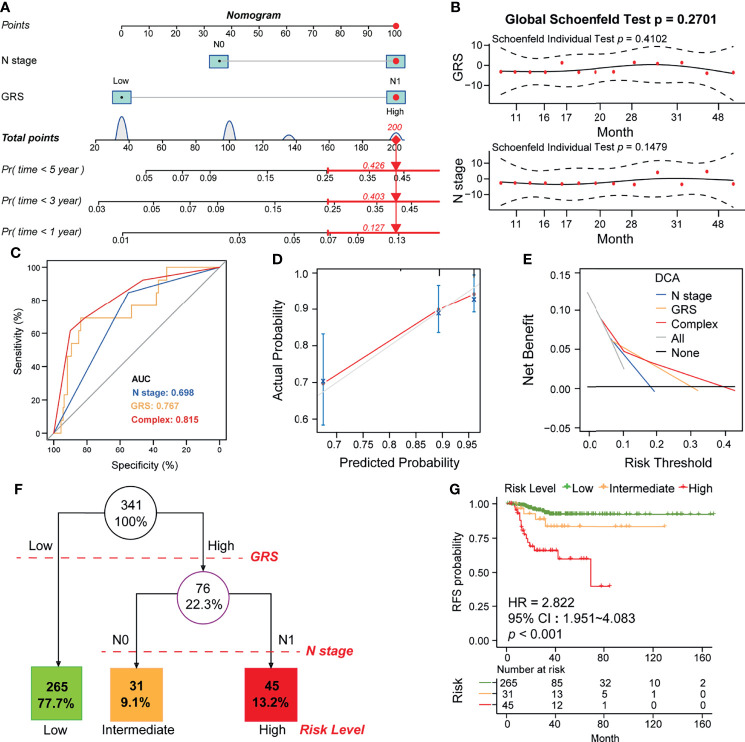
Construction of the GRS-based nomogram and clinical decision tree. **(A)** Construction of the nomogram based on GRS and N stage. An example of the use of the nomogram is shown in red. The risk of 1-year, 3-year, and 5-year recurrence is indicated by a red arrow. For example, patients with N0 and high GRS were used. **(B)** Residual analysis to test whether the nomogram holds. **(C)** Calibration analysis to test the predictive accuracy of the nomogram. **(D)** ROC analysis comparing the predictive sensitivity of the nomogram and N-stage alone to GRS. **(E)** DCA analysis comparing the net survival benefit of the nomogram, GRS and N-stage. **(F)** Construction of a clinical decision tree based on GRS and N stage. **(G)** K-M analysis to compare survival differences between different risk groups.

Since we confirmed that GRS and N-stage were independent risk factors for RFS in DTC (**Figure 7D**), we constructed an integrated model based on these two factors. To quantify the risk assessment for individual DTC patients, a nomogram was generated using GRS together with N-stage, and the red arrow shows an example (**Figure 8A**). According to the nomogram, when a DTC patient with high GRS and N-stage was N0, the risk of recurrence at 1 year, 3 years, and 5 years was 0.127, 0.403, and 0.426, respectively. The underlying proportional hazards assumptions of the nomogram model were verified using Schoenfeld residual tests (**Figure 8B**). Furthermore, we compared the value of the nomogram model to the GRS model and traditional clinicopathological features and found that the nomogram exhibited the most powerful capacity for RFS prediction (**Figure 8C**). Similarly, both calibration tests and DCA graphically illustrated that the nomogram model exhibited more accuracy in estimating patient recurrence than other parameters (**Figures 8D, E**).

Finally, to aid clinical decision-making, we constructed a recurrence decision tree with N-stage and GRS to optimize risk stratification. As shown in **Figure 8F**, three different risk subgroups were defined based on two primary components: patients with low GRS were defined as the “low risk” group, while patients with high GRS & N0 stage and high GRS & N1 stage were defined as “intermediate risk” and “high risk” groups, respectively. As shown in the K-M curve, RFS differed markedly between the three risk subgroups (**Figure 8G**).

## Discussion

The increasing prevalence and incidence of DTC each year makes it a worldwide health concern. Although DTC is an indolent disease associated with excellent overall survival, its high recurrence rate remains a challenge for current postoperative management. Since recurrent disease often necessitates remedial surgery, additional administration of radioactive iodine (RAI) and more intensive long-term surveillance (23), the ability to determine the risk of disease recurrence may be a more meaningful outcome for both patients and clinicians. In the present study, we are the first to construct an integrated model incorporating clinicopathological features and glycolysis-related gene signatures, which performed well in differentiating between low‐ and high‐risk cohorts and showed good performance for predicting RFS in patients with DTC.

Although several follow-up data suggest minimal differences in disease-specific survival (DSS) between total thyroidectomy and lobectomy, the extent of surgery has a definite impact on recurrence in DTC patients (24). In this study, all patients with DTC who were used for analysis underwent total thyroidectomy; however, approximately 10% of patients relapsed after surgery. Therefore, a larger surgical scope does not prevent recurrence and increases the likelihood of surgical complications. According to previous reports, age, ETE, LNM, tumor size and tumor multifocality were found to be risk factors for the recurrence of DTC (22, 25). Consistent with previous studies, we also found that stage, T stage and N stage were significantly associated with RFS in DTC patients; however, only N stage was an independent risk factor for RFS.

Emerging evidence has implied that predictive models based on gene signatures can quantify the risk of tumor recurrence, but few studies have attempted to apply them in DTC. Our previous studies confirmed that abnormal glycolysis plays an important role in promoting the invasion and metastasis of DTC (10, 12). Similarly, in the present study, we found that the glycolysis level in tumor tissues was significantly associated with poor prognosis in DTC patients. Through univariate/multivariate Cox, LASSO and ROC analyses, we demonstrated that the GRS could be an independent prognostic factor in DTC patients, as calculated using the gene expression signatures of four GRGs (ADM, CD44, MKI67 and TYMS). It has been reported that CD44, MKI67 and TYMS are overexpressed in many cancer types, including DTC, and regulate glucose metabolism by targeting different genes (26–29). CD44, a marker of cancer stem-like cells and epithelial-mesenchymal transition, was found to increase CREB phosphorylation and sustain the proliferation of thyroid cancer cells (27). However, the function of the remaining 3 genes in DTC remains unclear. In the mechanistic validation of this study, we found that all four GRGs participate in promoting cell proliferation and metastasis, which may be attributed to their function in remodeling the tumor immune microenvironment of DTC. Interestingly, similar results were found in another ten cancers, which indirectly illustrates that the prognostic assessment function of the four-GRG signature may be generalizable across cancer types.

Multivariate Cox regression analysis demonstrated that GRS was a fairly strong predictor of risk for overall recurrence and survival, even stronger than age and clinical stage. To quantify the risk assessment for individual patients, we generated a nomogram including GRS and N-stage. Calibration analysis revealed that the nomogram showed very close to accurate prediction of actual survival. In addition, ROC analysis revealed that the nomogram showed more stable and stronger predictive power at different time points during follow-up compared to any other individual variable. We combined clinicopathological characteristics to construct a decision tree to improve risk stratification and optimize existing clinical guidelines. In the decision tree, GRS is the primary determinant. However, in the second node, the decision tree demonstrated that risk stratification was improved if GRS was replaced with N stage. Together, the decision tree and multivariate Cox results suggest that the glycolytic gene signature is indeed a strong risk factor for e recurrence and survival in DTC patients.

Some limitations of our study should be acknowledged. First, this was a retrospective study, and therefore, the prognostic robustness and clinical utility of the glycolysis-related gene signature need to be further validated in larger prospective trials. Second, further experimental studies are needed to elucidate the biological functions underlying the GRS genes related to tumor glycolysis.

In summary, we established a novel glycolysis-related gene signature to discriminate patients with DTC who are at high risk of recurrence. Integrating this with clinicopathological features, we constructed a decision tree to optimize risk stratification for recurrence-free survival and a nomogram to quantify risk assessment for individual patients. The GRG signature-based model may help clinicians provide a prognosis for DTC patients with a high risk of recurrence after surgery and provide further personalized treatment to decrease the chance of relapse.

## Data Availability Statement

The original contributions presented in the study are included in the article/**Supplementary Material**. Further inquiries can be directed to the corresponding authors.

## Ethics Statement

This study is based on published or public datasets and does not include new data that require ethical approval or consent.

## Author Contributions

MW, PH, and SC conceived and planned the study design. MW, BW, PC, Q-mS, H-lT, D-jO-y, B-qH, ML, Z-eQ, NL, and H-yH performed formal analysis and data interpretation. MW and BW wrote the original draft. PH and SC provided critical revisions and contributed to the editing of the paper. All authors read and approved the final manuscript.

## Funding

This work was supported by the National Natural Science Foundation of China (grant numbers 81974423, 81902729), the Key Research and Development Program of Hunan Province (grant number 2019SK2031), the China Postdoctoral Science Foundation (grant number 2020M672517, 2021T140749), the Natural Science Foundation of Hunan Province (grant number 2020JJ5904), and the Xiangya Hospital Foundation for Young Scholars (grant number 2018Q01).

## Conflict of Interest

The authors declare that the research was conducted in the absence of any commercial or financial relationships that could be construed as a potential conflict of interest.

## Publisher’s Note

All claims expressed in this article are solely those of the authors and do not necessarily represent those of their affiliated organizations, or those of the publisher, the editors and the reviewers. Any product that may be evaluated in this article, or claim that may be made by its manufacturer, is not guaranteed or endorsed by the publisher.

## Supplementary Material

Supplementary Figure 1 Heatmap showing the expression of 175 GRGs in DTC tissues.

Supplementary Figure 2 Comparative analysis of the GRS level in different DTC subtypes. The clinical information was downloaded from the TCGA database. The GRS level was compared in DTC patients with different **(A)** age, **(B)** AJCC stage, **(C)** T stage, **(D)** lymph node metastasis status, and **(E)** histology grade, Student’s t-test, ns *p*>0.05, *p<0.05, ***p*<0.01, ****p*<0.001.

Supplementary Figure 3 Boxplot-typed visualization of GRGs mRNA level in follicular **(A)** and papillary thyroid cancers **(B)**. Student’s t-test, ns *p*>0.05, **p*<0.05, ***p*<0.01, ****p*<0.001.

Supplementary Figure 4 Biological processes in gene ontology (GO) and Kyoto Encyclopedia of Genes and Genomes (KEGG) pathway assessment of upregulated genes in high GRS DTC patients.

Supplementary Figure 5 K-M analysis comparing survival differences between different ESTIMATE score groups.

Supplementary Figure 6
**(A)** Mutation information and **(B)** mutation sites of the four GRGs in thyroid cancer.

Supplementary Figure 7 Protein–protein interaction network constructed using the four GRGs.

Supplementary Figure 8 Pie chart displaying the possible involvement of the four GRGs in 10 cancer-related pathways using GSCALite (http://bioinfo.life.hust.edu.cn/web/GSCALite/).

Supplementary Figure 9 The role of GRS score in overall survival **(A)** and recurrence free survival **(B)** among pan-cancer. OS, overall survival; RFS, recurrence free survival; HR, hazard ratio.

Supplementary Figure 10 The first, third- and fifth-year AUC **(A)** and calibration curves **(B)** of the GRS model.

## Abbreviations

DTC, differentiated thyroid cancer; TNM, tumor node metastasis stage; GRG, glycolysis-related genes; TCGA, the cancer genome atlas; GEO, gene expression omnibus; GRS, glycolysis-related prognostic risk score; PTC, papillary thyroid carcinoma; FTC, follicular thyroid carcinoma; LNM, lymph node metastasis; ETE, extrathyroidal extension; ENE, extranodal extension; ATA, the American thyroid association; THCA, thyroid cancer cohort; GSVA, gene set variation analysis; K-M, Kaplan-Meier; ROC, receiver operating characteristic; AUC, area under the ROC curve; DCA, decision curve analysis; logFC, log2 (fold change); HPA, Human Protein Atlas; DEGs, differentially expressed genes; EMT, epithelial-mesenchymal transition; BLCA, Bladder Urothelial Carcinoma; BRCA, Breast invasive carcinoma; ESCA, Esophageal carcinoma; HNSC, Head and Neck squamous cell carcinoma; KIRC, Kidney renal clear cell carcinoma; KIRP, Kidney renal papillary cell carcinoma; LIHC, Liver hepatocellular carcinoma; LUAD, Lung adenocarcinoma; LUSC Lung squamous cell carcinoma; STAD, Stomach adenocarcinoma; HR, hazard ratio; 95%CI, 95% confidence interval; DSS, disease specific survival.

## References

[1] La Vecchia C Malvezzi M Bosetti C Garavello W Bertuccio P Levi F. Thyroid Cancer Mortality and Incidence: A Global Overview. Int J Cancer (2015) 136:2187–95. doi: 10.1002/ijc.29251 25284703

[2] Lin P Guo Y-N Shi L Li XJ Yang H He Y. Development of a Prognostic Index Based on an Immunogenomic Landscape Analysis of Papillary Thyroid Cancer. Aging (Albany NY (2019) 11:480. doi: 10.18632/aging.101754 30661062 PMC6366981

[3] Zha T Wu H. Expression of Serum AMPD1 in Thyroid Carcinoma and its Clinical Significance. Exp Ther Med (2018) 15:3357–61. doi: 10.3892/etm.2018.5859 PMC5840934 29545855

[4] Park J Kim K Lim DJ Bae JS Kim JS. Male Sex Is Not an Independent Risk Factor for Recurrence of Differentiated Thyroid Cancer: A Propensity Score-Matching Study. Sci Rep (2021) 11:14908. doi: 10.1038/s41598-021-94461-5 34290341 PMC8295365

[5] Liu FH Kuo SF Hsueh C Chao TC Lin JD. Postoperative Recurrence of Papillary Thyroid Carcinoma With Lymph Node Metastasis. J Surg Oncol (2015) 112:149–54. doi: 10.1002/jso.23967 PMC5034820 26175314

[6] Zhang L Wang Y Li X Wang Y Wu K Wu J. Identification of a Recurrence Signature and Validation of Cell Infiltration Level of Thyroid Cancer Microenvironment. Front Endocrinol (Lausanne) (2020) 11:467–7. doi: 10.3389/fendo.2020.00467 PMC7390823 32793117

[7] Zhao H Zhang S Shao S Fang H. Identification of a Prognostic 3-Gene Risk Prediction Model for Thyroid Cancer. Front Endocrinol (Lausanne) (2020) 11:510–0. doi: 10.3389/fendo.2020.00510 PMC7423967 32849296

[8] Wu M Yuan H Li X Liao Q Liu Z. Identification of a Five-Gene Signature and Establishment of a Prognostic Nomogram to Predict Progression-Free Interval of Papillary Thyroid Carcinoma. Front Endocrinol (Lausanne) (2019) 10:790–0. doi: 10.3389/fendo.2019.00790 PMC6872544 31803141

[9] Park CH Song CM Ji YB Pyo JY Yi KJ Song YS. Significance of the Extracapsular Spread of Metastatic Lymph Nodes in Papillary Thyroid Carcinoma. Clin Exp Otorhinolaryngol (2015) 8:289–94. doi: 10.3342/ceo.2015.8.3.289 PMC4553362 26330926

[10] Huang P Mao LF Zhang ZP Lv WW Feng XP Liao HJ. Down-Regulated miR-125a-5p Promotes the Reprogramming of Glucose Metabolism and Cell Malignancy by Increasing Levels of CD147 in Thyroid Cancer. Thyroid (2018) 28:613–23. doi: 10.1089/thy.2017.0401 29634399

[11] Byun JK Choi YK Kang YN Jang BK Kang KJ Jeon YH. Retinoic Acid-Related Orphan Receptor Alpha Reprograms Glucose Metabolism in Glutamine-Deficient Hepatoma Cells. Hepatology (2015) 61:953–64. doi: 10.1002/hep.27577 25346526

[12] Huang P Chang S Jiang X Su J Dong C Liu X. RNA Interference Targeting CD147 Inhibits the Proliferation, Invasiveness, and Metastatic Activity of Thyroid Carcinoma Cells by Down-Regulating Glycolysis. Int J Clin Exp Pathol (2015) 8:309–18. PMC4348865 25755717

[13] Thakur S Daley B Gaskins K Vasko VV Boufraqech M Patel D. Metformin Targets Mitochondrial Glycerophosphate Dehydrogenase to Control Rate of Oxidative Phosphorylation and Growth of Thyroid Cancer. In Vitro In Vivo. Clin Cancer Res (2018) 24:4030–43. doi: 10.1158/1078-0432.CCR-17-3167 PMC6095745 29691295

[14] Lee N Jang W-J Seo JH Lee S Jeong C-H. 2-Deoxy-D-Glucose-Induced Metabolic Alteration in Human Oral Squamous SCC15 Cells: Involvement of N-Glycosylation of Axl and Met. Metabolites (2019) 9:188. doi: 10.3390/metabo9090188 PMC6780519 31533338

[15] Husain Z Huang Y Seth P Sukhatme VP. Tumor-Derived Lactate Modifies Antitumor Immune Response: Effect on Myeloid-Derived Suppressor Cells and NK Cells. J Immunol (2013) 191:1486–95. doi: 10.4049/jimmunol.1202702 23817426

[16] Zhang XP Chen Q Liu Q Wang Y Wang F Zhao ZM. Development and Validation of Glycolysis-Related Prognostic Score for Prediction of Prognosis and Chemosensitivity of Pancreatic Ductal Adenocarcinoma. J Cell Mol Med (2021) 25:5615–27. doi: 10.1111/jcmm.16573 PMC8184720 33942483

[17] Zhao X Liu Z Ren Z Wang H Wang Z Zhai J. Triptolide Inhibits Pancreatic Cancer Cell Proliferation and Migration *via* Down-Regulating PLAU Based on Network Pharmacology of Tripterygium Wilfordii Hook F. Eur J Pharmacol (2020) 880:173225. doi: 10.1016/j.ejphar.2020.173225 32464191

[18] Safran M Dalah I Alexander J Rosen N Stein TI Shmoish M. GeneCards Version 3: The Human Gene Integrator. Database (2010) 2018. doi: 10.1093/database/baq020 PMC2938269 20689021

[19] Zou C Yuan C Ye J Liu Z Gao X Piao X. Identification and Validation of a Ten-Gene Set Variation Score as a Diagnostic and Prognostic Stratification Tool in Hepatocellular Carcinoma. Am J Transl Res (2020) 12:5683–95. PMC7540149 33042448

[20] Li T Fan J Wang B Traugh N Chen Q Liu JS. TIMER: A Web Server for Comprehensive Analysis of Tumor-Infiltrating Immune Cells. Cancer Res (2017) 77:e108–10. doi: 10.1158/0008-5472.CAN-17-0307 PMC6042652 29092952

[21] Wang H Wu X Chen Y. Stromal-Immune Score-Based Gene Signature: A Prognosis Stratification Tool in Gastric Cancer. Front Oncol (2019) 9:1212. doi: 10.3389/fonc.2019.01212 31781506 PMC6861210

[22] Yan H Zhou X Jin H Li X Zheng M Ming X. A Study on Central Lymph Node Metastasis in 543 Cn0 Papillary Thyroid Carcinoma Patients. Int J Endocrinol (2016) 2016:1878194. doi: 10.1155/2016/1878194 27127507 PMC4834155

[23] Filetti S Durante C Hartl D Leboulleux S Locati LD Newbold K. Thyroid Cancer: ESMO Clinical Practice Guidelines for Diagnosis, Treatment and Follow-Updagger. Ann Oncol (2019) 30:1856–83. doi: 10.1093/annonc/mdz400 31549998

[24] Gartland RM Lubitz CC. Impact of Extent of Surgery on Tumor Recurrence and Survival for Papillary Thyroid Cancer Patients. Ann Surg Oncol (2018) 25:2520–5. doi: 10.1245/s10434-018-6550-2 PMC6070400 29855833

[25] Lan X Sun W Zhang H Dong W Wang Z. Zhang T. A Meta-Analysis of Central Lymph Node Metastasis for Predicting Lateral Involvement in Papillary Thyroid Carcinoma. ORG Head Neck Surg (2015) 153:731–8. doi: 10.1177/0194599815601412 26307575

[26] Zhang Z Xu T Qin W Huang B Chen W Li S. Upregulated PTPN2 Induced by Inflammatory Response or Oxidative Stress Stimulates the Progression of Thyroid Cancer. Biochem Biophys Res Commun (2020) 522:21–5. doi: 10.1016/j.bbrc.2019.11.047 31735335

[27] De Falco V Tamburrino A Ventre S Castellone MD Malek M Manié SN. CD44 Proteolysis Increases CREB Phosphorylation and Sustains Proliferation of Thyroid Cancer Cells. Cancer Res (2012) 72:1449–58. doi: 10.1158/0008-5472.CAN-11-3320 22271686

[28] Hossain MA Asa TA Rahman MM Uddin S Moustafa AA Quinn JMW. Network-Based Genetic Profiling Reveals Cellular Pathway Differences Between Follicular Thyroid Carcinoma and Follicular Thyroid Adenoma. Int J Environ Res Public Health (2020) 17(4):1373. doi: 10.3390/ijerph17041373 PMC7068514 32093341

[29] Gao R Li D Xun J Zhou W Li J Wang J. CD44ICD Promotes Breast Cancer Stemness *via* PFKFB4-Mediated Glucose Metabolism. Theranostics (2018) 8:6248–62. doi: 10.7150/thno.28721 PMC6299690 30613295

