# Supplementary figures and images for: A Prognostic Model of Differentiated Thyroid Cancer Based on Up-Regulated Glycolysis-Related Genes
Source: Front Endocrinol (Lausanne). 2022 Apr 22;13:775278. doi: 10.3389/fendo.2022.775278 (PMC9072639; doi:10.3389/fendo.2022.775278)

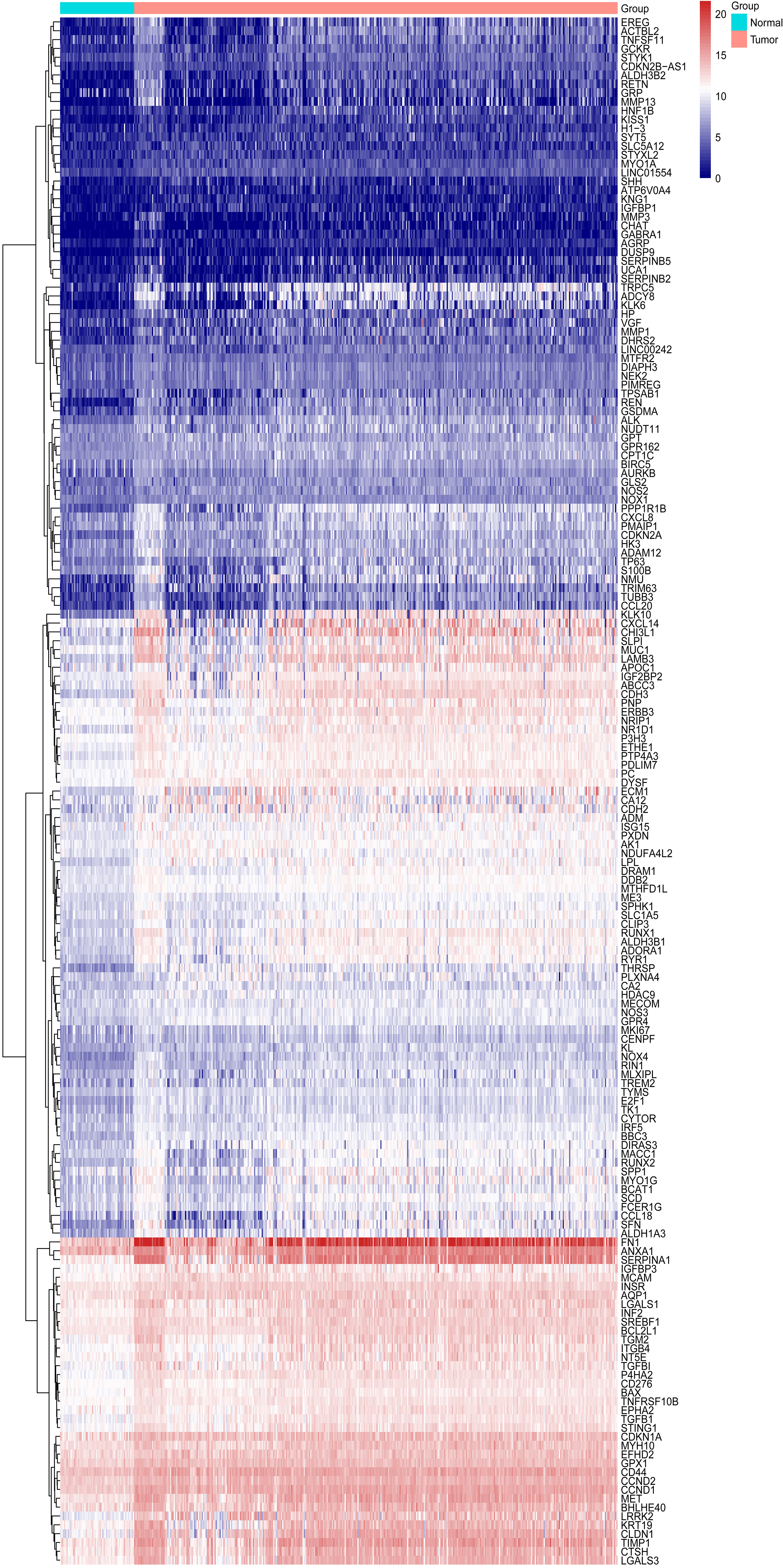

Supplement: Supplementary Figure 1 — Heatmap showing the expression of 175 GRGs in DTC tissues. [file Image_1.jpeg]

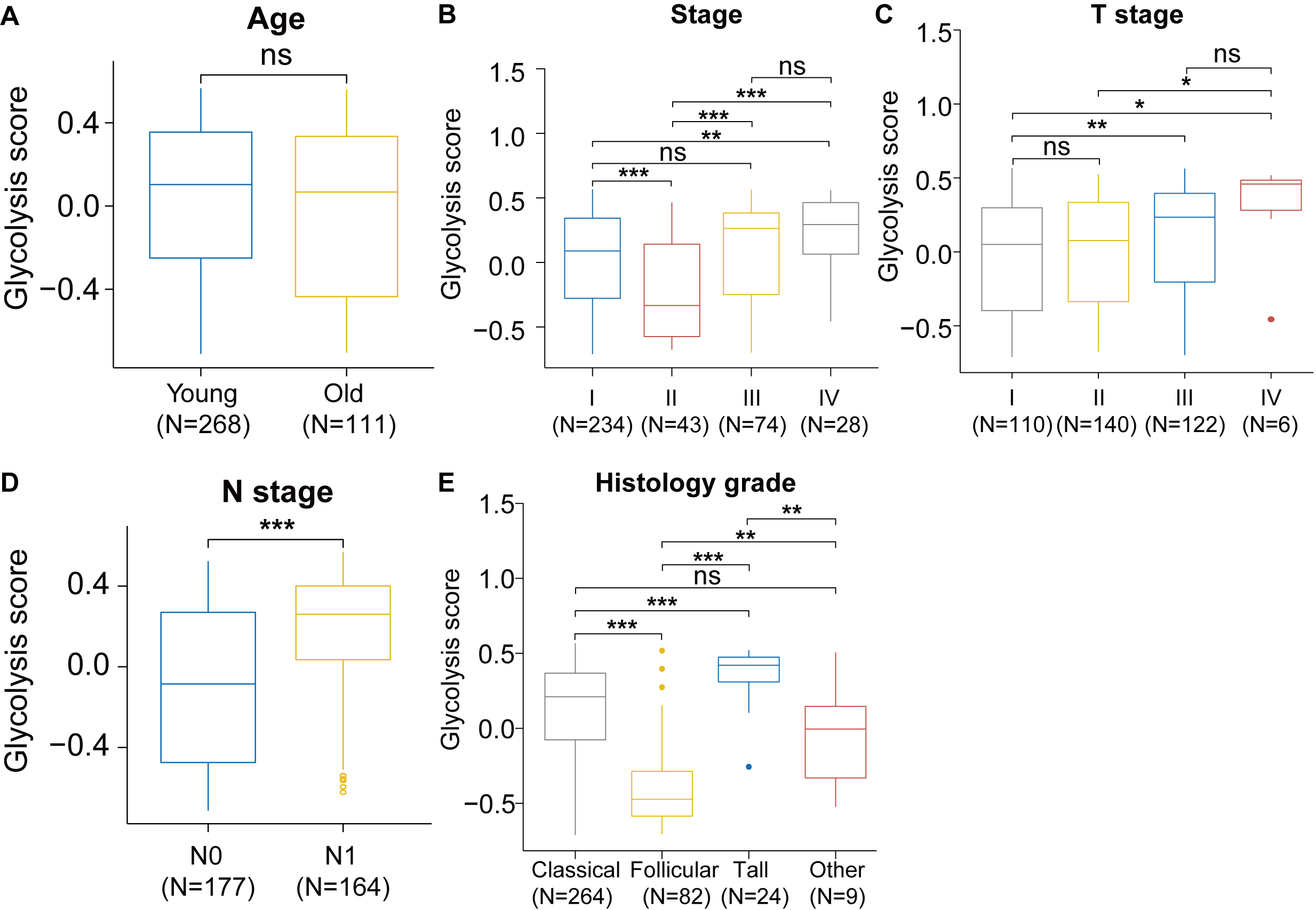

Supplement: Supplementary Figure 2 — Comparative analysis of the GRS level in different DTC subtypes. The clinical information was downloaded from the TCGA database. The GRS level was compared in DTC patients with different (A) age, (B) AJCC stage, (C) T stage, (D) lymph node metastasis status, and (E) histology grade, Student’s t-test, ns p>0.05, *p<0.05, **p<0.01, ***p<0.001. [file Image_2.tif]

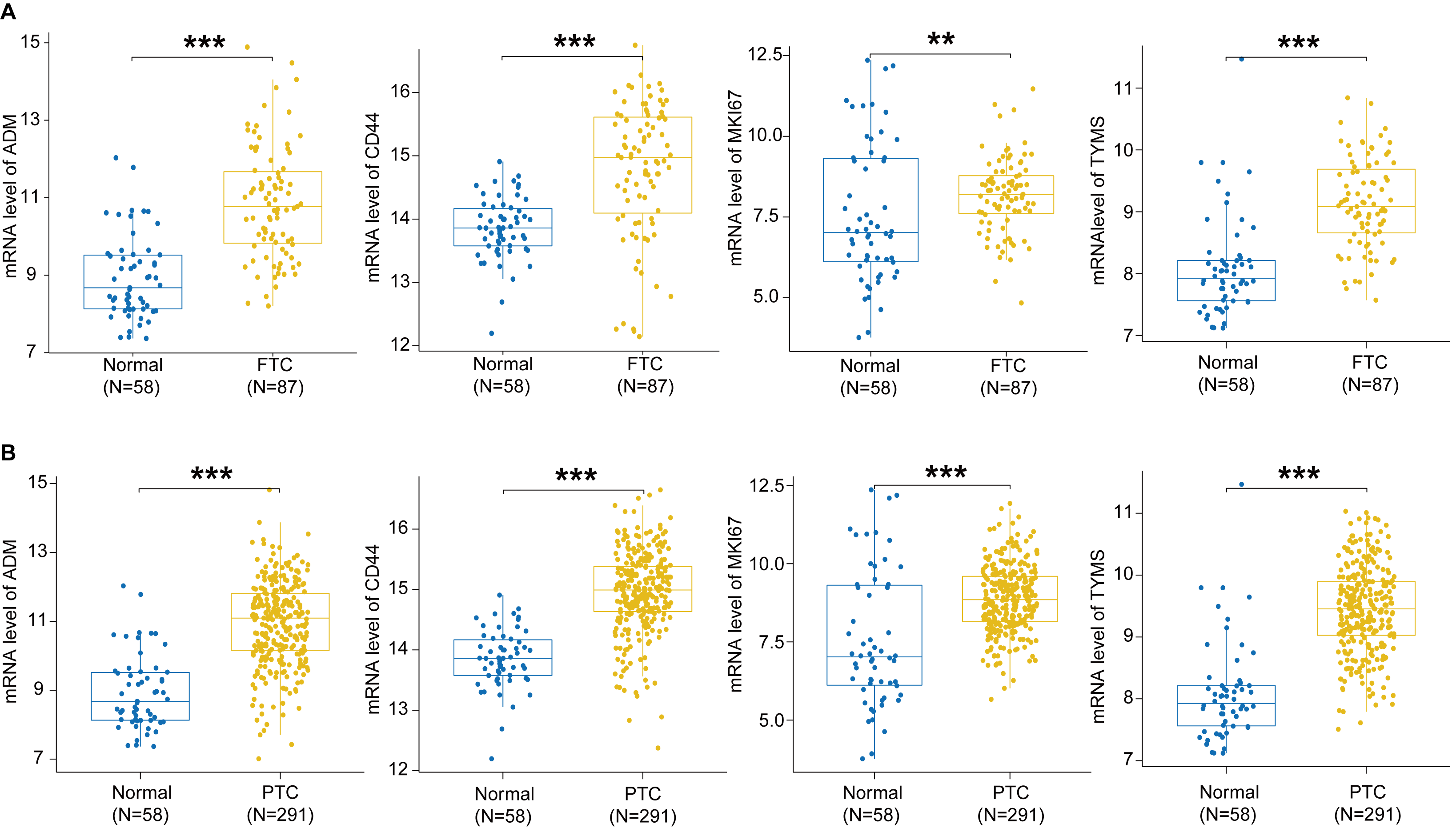

Supplement: Supplementary Figure 3 — Boxplot-typed visualization of GRGs mRNA level in follicular (A) and papillary thyroid cancers (B). Student’s t-test, ns p>0.05, *p<0.05, **p<0.01, ***p<0.001. [file Image_3.tif]

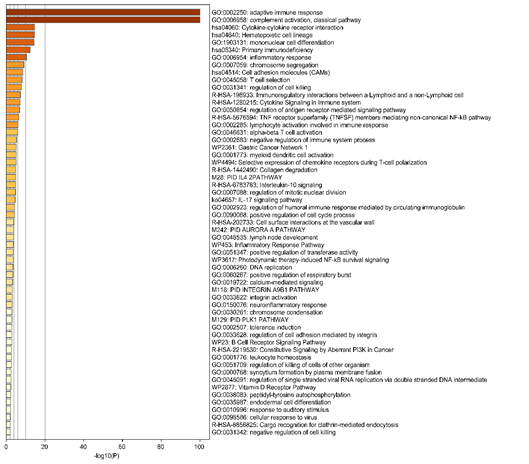

Supplement: Supplementary Figure 4 — Biological processes in gene ontology (GO) and Kyoto Encyclopedia of Genes and Genomes (KEGG) pathway assessment of upregulated genes in high GRS DTC patients. [file Image_4.tif]

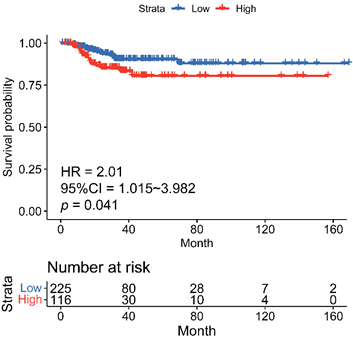

Supplement: Supplementary Figure 5 — K-M analysis comparing survival differences between different ESTIMATE score groups. [file Image_5.tif]

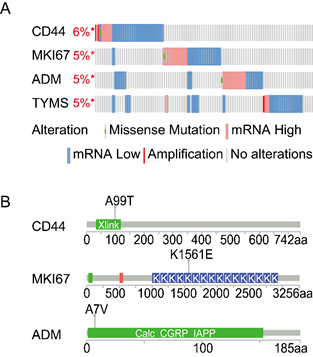

Supplement: Supplementary Figure 6 — (A) Mutation information and (B) mutation sites of the four GRGs in thyroid cancer. [file Image_6.tif]

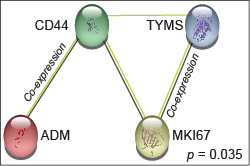

Supplement: Supplementary Figure 7 — Protein–protein interaction network constructed using the four GRGs. [file Image_7.tif]

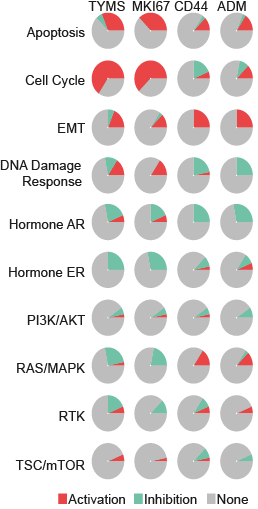

Supplement: Supplementary Figure 8 — Pie chart displaying the possible involvement of the four GRGs in 10 cancer-related pathways using GSCALite (http://bioinfo.life.hust.edu.cn/web/GSCALite/). [file Image_8.tif]

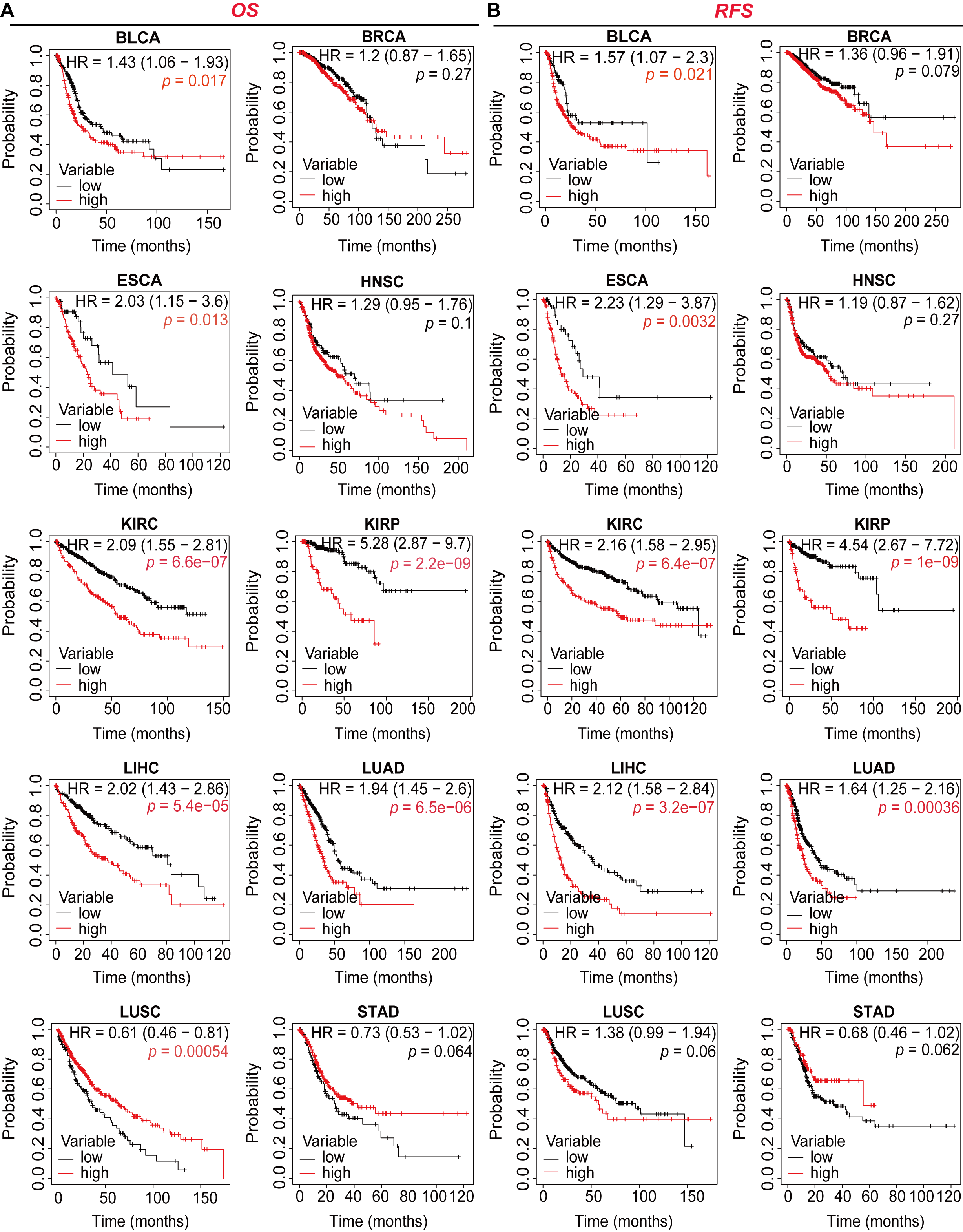

Supplement: Supplementary Figure 9 — The role of GRS score in overall survival (A) and recurrence free survival (B) among pan-cancer. OS, overall survival; RFS, recurrence free survival; HR, hazard ratio. [file Image_9.tif]

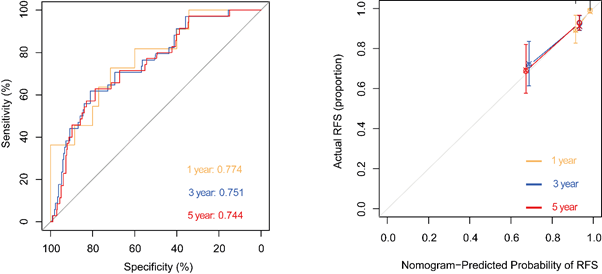

Supplement: Supplementary Figure 10 — The first, third- and fifth-year AUC (A) and calibration curves (B) of the GRS model. [file Image_10.tif]
